# Alligators in the abyss: The first experimental reptilian food fall in the deep ocean

**DOI:** 10.1371/journal.pone.0225345

**Published:** 2019-12-20

**Authors:** Craig Robert McClain, Clifton Nunnally, River Dixon, Greg W. Rouse, Mark Benfield

**Affiliations:** 1 Louisiana Universities Marine Consortium, Chauvin, LA, United States of America; 2 Department of Biology, University of Louisiana, Lafayette, LA, United States of America; 3 Scripps Oceanography, UC San Diego, La Jolla, CA, United States of America; 4 Department of Oceanography and Coastal Sciences, College of the Coast and Environment, Louisiana State University, Baton Rouge, LA, United States of America; Museum National d'Histoire Naturelle, FRANCE

## Abstract

The high respiration rates of the deep-sea benthos cannot be sustained by known carbon supply pathways alone. Here, we investigate moderately-sized reptilian food falls as a potential alternative carbon pathway. Specifically, three individual carcasses of *Alligator mississippiensis* were deployed along the continental slope of the northern Gulf of Mexico at depths of ~2000m in early 2019. We posit the tough hide of alligators would impeded scavengers by limiting access to soft tissues of the alligator fall. However, the scavengers began consuming the food fall 43 hours post-deployment for one individual (198.2cm, 29.7kg), and the carcass of another individual (175.3 cm, 19.5kg) was completely devoid of soft tissue at 51 days post-deployment. A third individual (172.7cm, 18.5kg) was missing completely after 8 days, with only the deployment harness and weight remaining drug 8 meters away, suggesting a large elasmobranch scavenger. Additionally, bones recovered post-deployment reveal the first observations of the bone-eating *Osedax* in the Gulf of Mexico and are confirmed here as new to science. The findings of this study indicate the quick and successful utilization of terrestrial and aquatic-based carbon food sources in the deep marine environment, though outcome variability may be high.

## Introduction

On the deep ocean floor, devoid of light, photosynthetically active radiation is nonexistent. Thus, in this ecosystem, the largest on Earth, primary production is virtually absent [1]. This lack of extensive carbon creates an energy-deprived system where ecological and evolutionary processes are both driven and constrained by carbon supply [1]. Much of this carbon supply is delivered through particulate organic carbon (POC) derived from primary production produced on the ocean’s surface [2]. However, benthic community respiration rates indicate that this carbon supply alone is unable to meet community demand[3]. The discrepancy between carbon supply and demand cannot be explained by seasonal variance in POC delivery, thus an alternate supply pathway must account for the difference. Alternative pathways such as chemosynthesis provide just a tiny fraction of total ocean production (0.02–0.03%) and a small percentage (3%) of carbon flux in food-limited deep-sea systems [4]. This leaves a carbon deficit.

The remains of large plants, algae, and animals arriving as bulk food parcels create areas of intense organic enrichment and may account, in part, for the missing carbon need [5–10]. Research has focused on naturally occurring and experimentally deployed wood and plant remains [7, 10, 11], cameras baited with animal carcasses [12–14], and chance occurrences of or deployed intact vertebrate carcasses on the deep-sea floor [5, 6, 15–24]. Prior work has shown these food-falls play an important role in deep-sea diversity; many of these large food falls on the deep-sea floor host highly diverse and endemic suites of species [5–7, 15–24]. In addition, these represent significant carbon transport highways into the deep oceans. During Typhoon Morakot, wood was estimated to carry a total of 4*10^12^ g of organic carbon into the oceans, nearly 25% of the total annual riverine discharge of organic carbon in Northwest Pacific Ocean [25]. On the deep-sea floor, a single wood fall can enrich sedimentary organic carbon by >25% even after several years. [10] Estimates of total carbon delivered to the deep-sea in the form of large carcasses range from 15–17% of total carbon exported for large mesopelagic fish [26] or 325 years of background POC flux for cetacean falls [27].

Prior research on animal falls has primarily focused on whale falls [5–7, 15–24]. A whale fall's substantial size and lipid-rich skeleton factor together to create a long-lasting food source on the deep-sea floor which contributes to high endemicity, community assembly and persistence, and ultimately enhanced biodiversity [28]. Thus, non-mammalian and smaller animal food falls may not be able to host fully developed deep-sea communities [6, 17, 28]. Research on dolphin [27], whale shark carcass [28], and mobulid ray [28] carcasses suggest that these smaller food-fall communities never proceed past a scavenger phase to reach a fully developed “whale fall” community.

Before the age of large marine mammals, large marine reptiles dominated the oceans. During the Mesozoic Era, rising to dominance in the Triassic and Jurassic periods, ichthyosaurs, plesiosaurs, and nothosaurs represented a diverse group of large marine predators. For example, the ichthyosaur *Shonisaurus sikanniensis* may have reached lengths of up to 21 meters in the Late Jurassic [29]. *Plesiosaurus* is estimated to have been 12–15 meters in length [30] and *Nothosaurus zhangi* 3–5 meters [31]. The sunken carcasses of these massive marine reptiles hosted diverse and endemic invertebrate communities and may have contributed significantly to the deep-sea carbon budget [32]. During the Middle Eocene, fossilized limpets are found in close association with bones of fossil leatherback turtles [33]. Mollusks also occur on fossilized ichthyosaur and plesiosaur remains [34]. Prehistoric ichthyosaur falls are known to support communities similar in ecology and succession to modern whale falls, supporting a wide array of macro- and micro-invertebrates [35, 36].

In the modern oceans, the 24 species of crocodilians may may fulfill an important ecological role into the deep oceans by serving as the closest modern analog of prehistoric marine reptile food falls. The group is geographically broad and concentrated in coastal regions and major river drainages that would facilitate carbon connections with marine systems [37]. Some crocodilian species also frequent marine habitats during migrations. For example, the estuarine crocodile, *Crocodylus porosus* is known to ride surface ocean currents to facilitate long-distance travel [38] well over 400 km in coastal saline waters of Australia [39]. In addition, non-threatened and endangered species, such as caymans and the American alligator, can reach total populations sizes well over million individuals [37] representing a considerable carbon pool.

Here we focus our study on the American alligator, *Alligator mississippiensis*. Both live individuals and carcasses of alligators are frequent on beaches and in coastal surf [40–45]. A 3-meter individual came ashore at Folly Beach, South Carolina in 2014 [46] and in 2016 a carcass of a 4-meter individual washed up on a beach in Galveston, Texas [47]. *A*. *mississippiensis* individuals are also common inhabitants on barrier islands [48, 49]. These individuals of *A*. *mississippiensis* may be easily carried offshore by major rivers or during large storm events, tropical storms, and hurricanes [50]. Live *A*. *mississippiensis* have been observed 63 kilometers offshore [45]. During the 2011 Mississippi flood event, several dead alligators were observed in the mouth of Atchafalaya River.

We deployed three individuals of *Alligator mississippiensis* at three 2000-meter sites in the deep Gulf of Mexico with observations from 24 hours to 51 days after deployment (Table 1). We hypothesize that the tough hide of alligators may prove difficult for scavengers, thus prolonging carbon delivery, and be unconducive to community assembly typically observed on whale falls.

**Table 1:** Details of deployment sites, observation dates, and carcass sizes for alligator falls in the Gulf of Mexico.

Site	Latitude	Longitude	ID	Length (cm)	Estimated Mass (kg)	Depth (m)	Date Deployed	Time Deployed	Date Observed	Time Observed	Time Since Deployment
WF1	27.1356°	-89.9269°	A1	198.2	29.7	2181	14-February-2019	1536	15-Feb-2019	1205	20:29
									16-Feb-19	1039	43:13
WF2	27.3126°	-88.9270°	A2	175.3	19.5	2034	20-February-2019	1743	12-April-19	1754	51d 00:11
WF3	28.1032°	-88.4516°	A3	172.7	18.5	1996	15-April-2019	1821	23-April-19	1205	8d 06:16

## Methods

*A*. *mississipiensis* is not endangered or threatened across its range in Louisiana. *A*. *mississippiensis* is a intensively managed species in Louisiana and individuals were obtained through Louisiana state procedures. Three carcasses of *A*. *mississippiensis* for the experiment came from the Louisiana Department of Wildlife and Fisheries, Alligator Management Program (LDWF Tags 898309, 898310, 898321). *A*. *mississippiensis* were frozen immediately post-mortem for later deployment. Calculated length of *A*. *mississippiensis* was measured as snout to tip of tail. For deployment on the sea floor with a remotely operated vehicle (ROV), each *A*. *mississippiensis* had a harness tied around the fore and hind limbs and included a handle for lifting by the manipulator arm. A 20 kg weight attached to the underside of the harness anchored the individual in place on the seafloor. The harness consisted of Continental Western Corporation (CWC) Blue Steel ½” line with a tensile strength of 6600 pounds (2994 kg).

**Fig 1:**
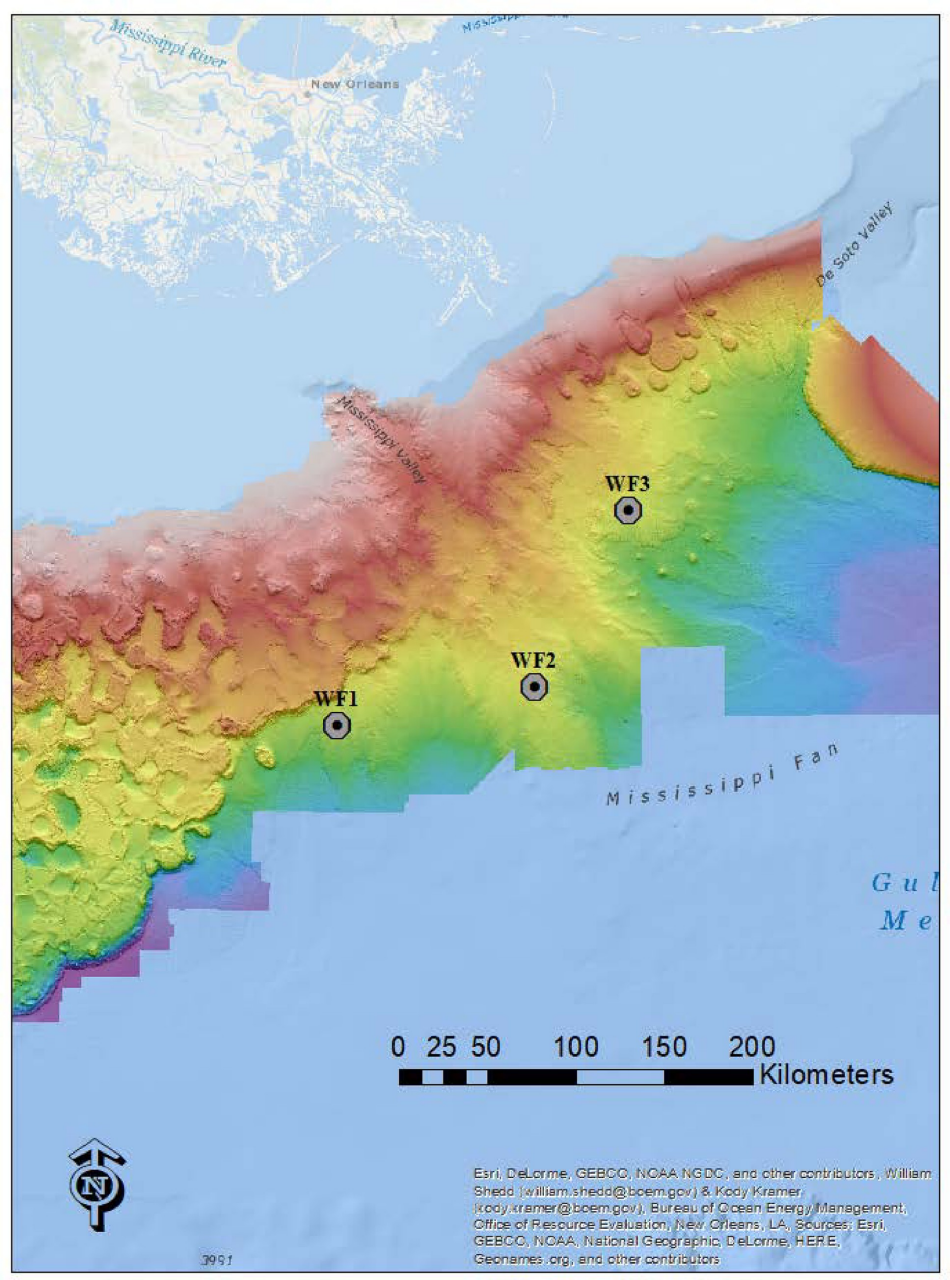
Map of the three alligator fall study sites in the northeast Gulf of Mexico.

*A*. *mississippiensis* were placed at three individual sites of similar depths along the northern Gulf of Mexico continental slope using the Oceaneering ROV *Global Explorer* on two cruises aboard the R/V *Pelican* in early 2019 (Fig 1, Table 1). No specific permissions were required for these locations and scientific research in the deep sea is permitted in the U.S. EEZ and international waters in areas non-designated as marine sanctuaries or otherwise protected.

*A*. *mississippiensis* arrived on the bottom via a work basket that the ROV accessed to remove them for deployment. Individuals were marked with a weighted float that enabled locating the experiments with sonar upon return.

Observation of the individuals was made by the ROV on subsequent dives as noted in Table 1. Video observations were made by allowing the ROV to land on either side of the individual and observing scavenger aggregations for 5–10 minutes each. Upon return to Alligator 2 at 51 days, post-deployment bones were recovered in a net sealed on the sea floor.

*A*. *mississippiensis* weights were estimated from length using allometric scaling equation based on Louisiana individuals of *A*. *mississippiensis* [51].

$$weight\left(kg\right)=1.35–0.0378*length\left(cm\right)+0.0000046*length{\left(cm\right)}^{3}$$

To estimate the total *A*. *mississippiensis* mass lost due to total scavenging of soft tissue, we assumed bone mass was 12.46% of the total individual weight based on published relationships [52].

Some bones were fixed and preserved in 95% ethanol and then examined for specimens of *Osedax*. These were extracted, photographed and subsampled for DNA extraction. Mitochondrial cytochrome *c* oxidase I (COI) sequence was amplified and sequenced for one specimen using the primers polyLCO and polyHCO [53]. The voucher specimen is deposited at the Scripps Institution of Oceanography Benthic Invertebrate Collection (SIO-BIC A10731). The COI sequence (GenBank MN258704) was compared to the available *Osedax* sequences using methods as outlined previously [54].

## Results

### Alligator 1

**Fig 2:**
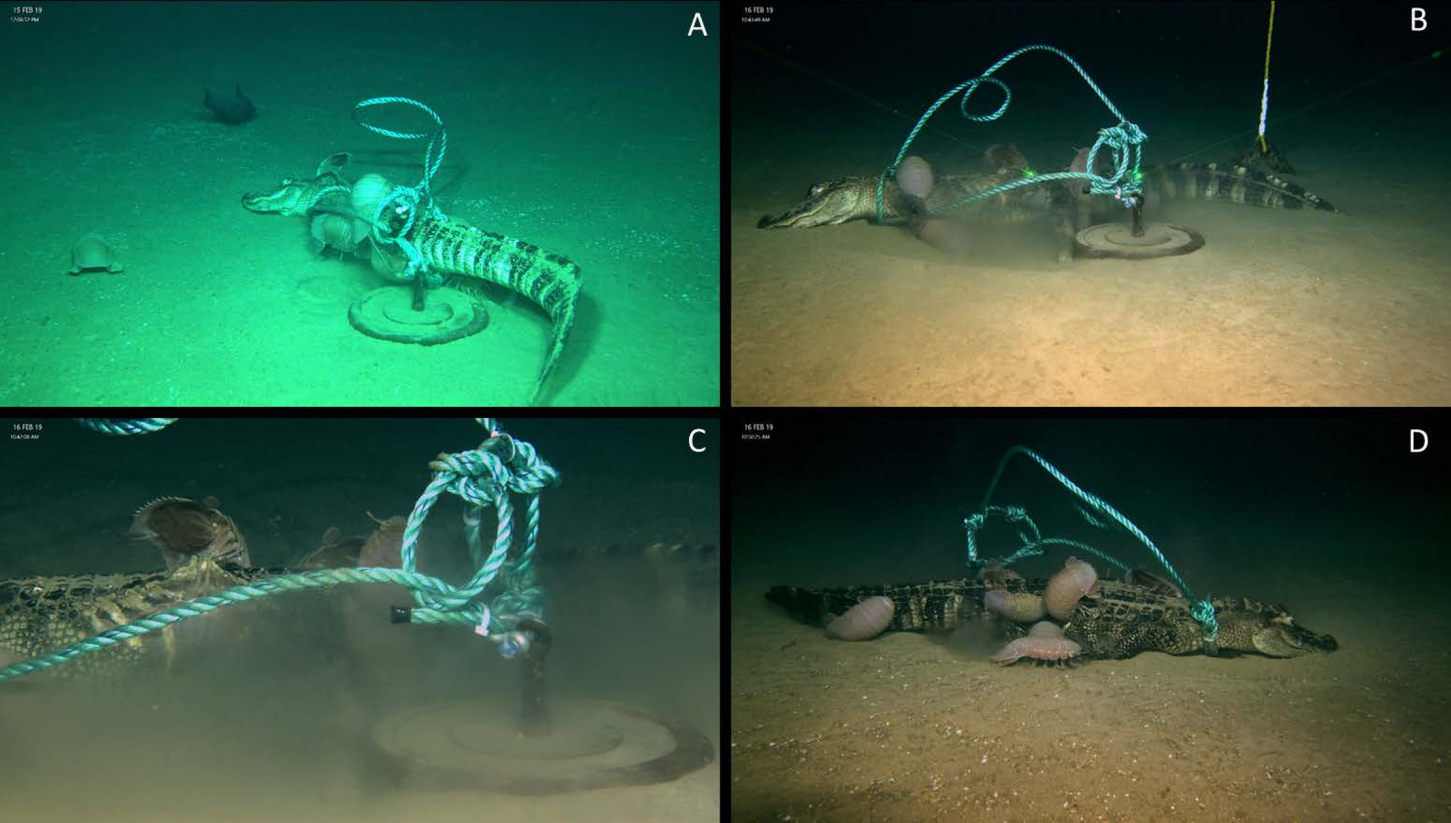
Alligator 1 deployed at WF1 during February 2019. A) *B*. *giganteus* feeding and in the vicinity a Macrourid after one day B) Left lateral view showing pit excavated by *B*. *giganteus* after two days C) *B*. *giganteus* visibly inside the rib cage of the alligator after two days D) Right lateral view of alligator with more *B*. *giganteus* feeding.

The individual was deployed on the bottom at 2180 meters at Site 1 on 14 February 2019 at 15:36. First observation of the alligator fall occurred at 15 February 2019 at 12:05. Nine *Bathynomus giganteus* were scavenging on the food fall (Fig 2). *B*. *giganteus* individuals fed at several points on the carcass including: two on the right lateral head, one on the the left lateral abdomen, two individuals near the intersection of the left lateral humerus and scapula, one individual on the dorsal side near the thoracic vertebra, one individual on the right lateral ilium, and one on the ventral side of the caudal vertebra at the base of the tail. A ninth individual occurred in the sediment approximately one meter away from the alligator fall. Additionally, one Macrourid (*Nezumia* sp.) swam around the food fall but never came closer than approximately one meter. Small amphipods, approximately 1–2 centimeters in length, were observed around the abdominal opening created by *B*. *giganteus*.

The second observation of the alligator fall occurred at 15 February 2019 at 13:57. An unidentified Oegospid squid hovered near the alligator fall. At the same time, a Macrourid individual was feeding on the carcass. An additional individual of *B*. *giganteus* was swimming toward the alligator fall.

**Fig 3:**
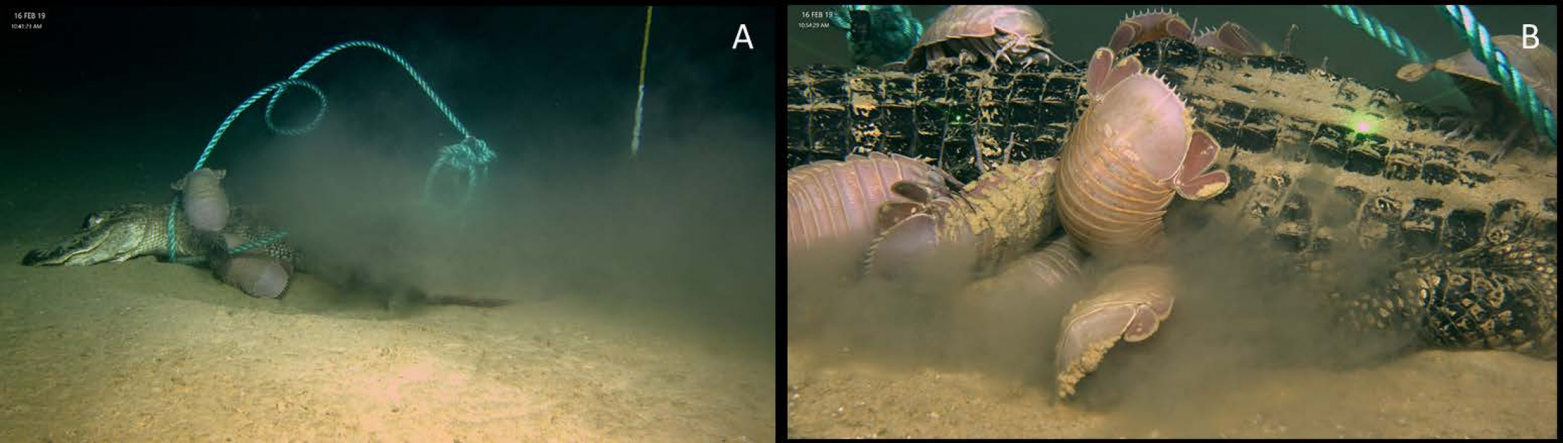
Bioturbation generated by feeding activities of *B*. *giganteus* A) left lateral view of sediment kicked up by feeding activities B) tightly packed *B*. *giganteus* around the dermal opening excavate under the alligator as they leverage for space.

The third observation of the alligator fall occurred on 16 February 2019 at 10:37. Thirteen total *B*. *giganteus* were observed feeding on the alligator fall. The left lateral thoracic ribs were exposed. No *B*. *giganteus* were seen feeding on the head. At least two *B*. *giganteus* were feeding wholly inside the right lateral area posterior to the thoracic ribs. Two individuals were continuing to feed near the intersection of the left lateral humerus and scapula. Two additional isopods were feeding in the region posterior to the abdomen and while not wholly inside were inserted into an additional second opening in the skin. One individual was located near the ventral side of the caudal vertebra at the base of the tail. Four individuals of *B*. *giganteus* were feeding in a third opening into the right lateral side just posterior of the thoracic ribs. An individual of the scavenging amphipod of *Eurythenes grillus* was also observed feeding in this region. A fifth individual of *B*. *giganteus* was observed near this area before swimming away and returning to the left lateral side. An additional *B*. *giganteus* was observed on the right lateral tail. Two individuals were seen swimming sporadically and then crashing into sediment. Beating of the book gills during feeding produced currents and, along with general movements of *B*. *giganteus* near the food fall, generated considerable sediment disturbance (Fig 3).

### Alligator 2

The individual was deployed on the bottom at 2034 meters at Site 2 on 20 February 2019 at 17:43. At 17:59, a single individual Macrourid swam to the alligator fall and investigated.

**Fig 4:**
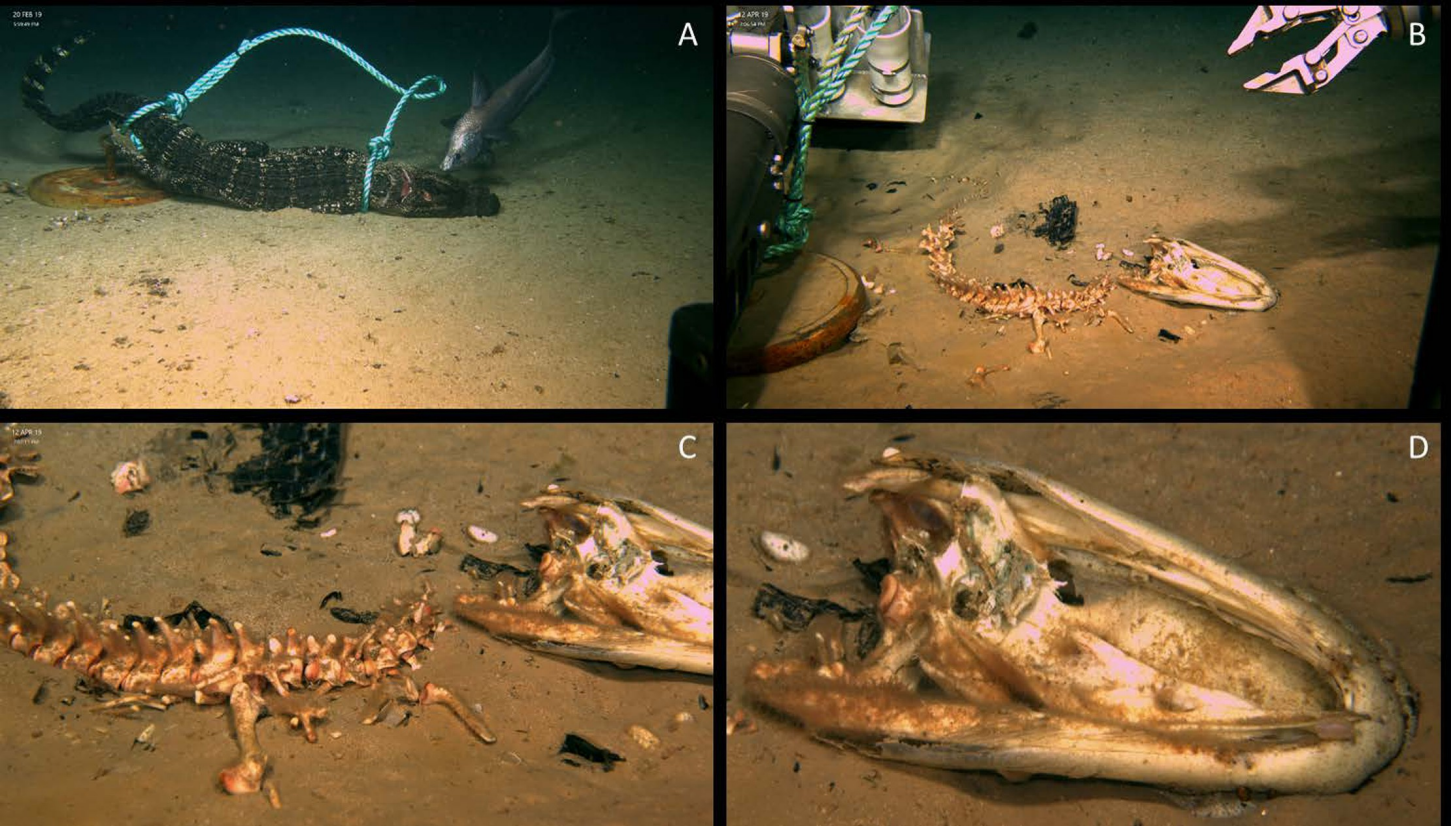
Alligator 2 deployed at WF2 during February 2019. A) Macrourid investigating alligator carcass on day of deployment B) Bones of alligator 2 51 days after deployment resting next to rope and weight C) Vertebrae and for legs of alligator 2 upon investigation 51 days since deployment D) Skull of alligator 2 with red hue of filamentous *Osedax* seen on lower jaw.

Subsequent observation of the food fall occurred 53 days later on 12 April 2019. At this time, the food fall was comprised of bones with all soft tissues completely absent. However, soft tissue remained on parts of the alligator skull and particularly the lower mandible. The alligator skull was also turned over and laying on its dorsal surface. Alligator scutes and hide were seen scattered across the food fall area and a sediment depression had formed under the alligator fall. *Osedax* sp., denoted by reddish hue seen in video, were observed forming dense patches across the alligator bones, particularly the vertebral column. These represented *Osedax* females with well-developed ovaries and oocytes within the bone. No dwarf males could be located in the tubes of the females examined. Analysis of the COI sequence obtained reveals that this is *Osedax* falls within the clade known as Clade II or ‘nudepalps’ [54]. It was ~14% divergent (uncorrected distance) from the nearest known *Osedax*, *O*. *crouchi* from Antarctica and *O*. *lonnyi* from California, and so is a new undescribed species, which will be named in due course (S1 Fig, Rouse in prep). Sediment around the food fall appeared to be highly disturbed and of a notable color difference (Fig 4).

### Alligator 3

**Fig 5:**
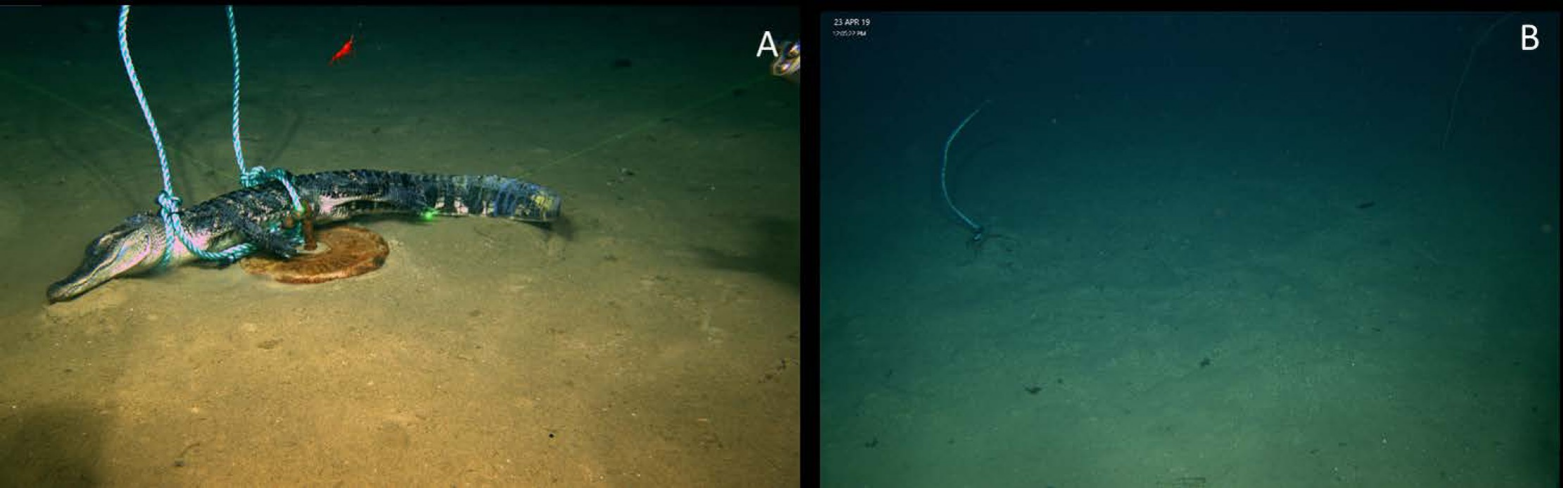
Alligator 3 deployed at WF3 during April 2019. A) resting on sea floor after deployment B) drag marks, weight and disturbed sediment was all that remained of alligator 3 in the 8 days since deployment.

The individual was deployed on the bottom at 1996 meters at Site 3 on 15 April 2019 at 18:21. Subsequent observation of the deployment site occurred 8 days later on 23 April 2019. At this time, although the marking float and weight remained, the food fall was absent. At a distance of 8.3 meters away, the 20.4 kg anchor weight, shackle, and part of the polypropylene line used to secure the alligator to the weight on the seafloor were discovered (Fig 5). Observations of the disturbed seafloor between the marking float and the anchor weight suggest at least the anchor weight, and potentially the food fall, was dragged to its new location.

## Discussion

Successional stages of large cetacean food falls have been described by Smith and colleagues [17, 55]. The first stage is the “mobile-scavenger stage” occurring on a time scale of four months to two years, depending on carcass size. During this stage, scavengers or necrophages ranging in size from small amphipods to large elasmobranchs are attracted to the food fall and devour the soft tissue of the carcass extremely quickly, at rates of 40–60 kg day^-1^. There may be succession within this stage in terms of scavenger body size as there are progressively smaller bits of tissue left to exploit on the carcass. The second stage is the “enrichment-opportunist” stage, where macrofauna (especially polychaetes and crustaceans) colonize the bones and organically enriched sediments surrounding the carcass. Although notable is that these successional stages may often overlap [56].

While densities of these communities are high and composed of both background and endemic fauna, overall diversity is low. The exact duration of this stage is unknown, but evidence suggest the time is fewer than two years. The third successional stage is the “sulfophilic stage”. Chemoautotrophic bacteria oxidizing sulfur from lipid breakdown within the bones form the base of a trophically complex and highly diverse community. This community includes chemoautotrophs and species with chemoautotrophic endosymbionts, bone-lipid consumers, and predators able to tolerate the highly sulfophilic environment. It is important to note that during this stage, the large carcasses host a predominantly chemoautotrophic community, while small carcasses have a predominantly sulfur-tolerant but not necessarily chemoautotrophic community. This stage can last for upwards of multiple decades. Last is the “reef stage”, in which the bones extracted of organic material serve as hard substrate for suspension feeders. However, recent work by Lundsten et al. [22] found little evidence of these four successional stages and especially the sulfophilic stage in juvenile whale falls, possibly owing to presence of *Osedax*, in Monterey Canyon experimental whale falls. These whale falls also were primarily of species found at the whale-falls regularly observed at non-background, non-whale fall sites [22].

**Fig 6:**
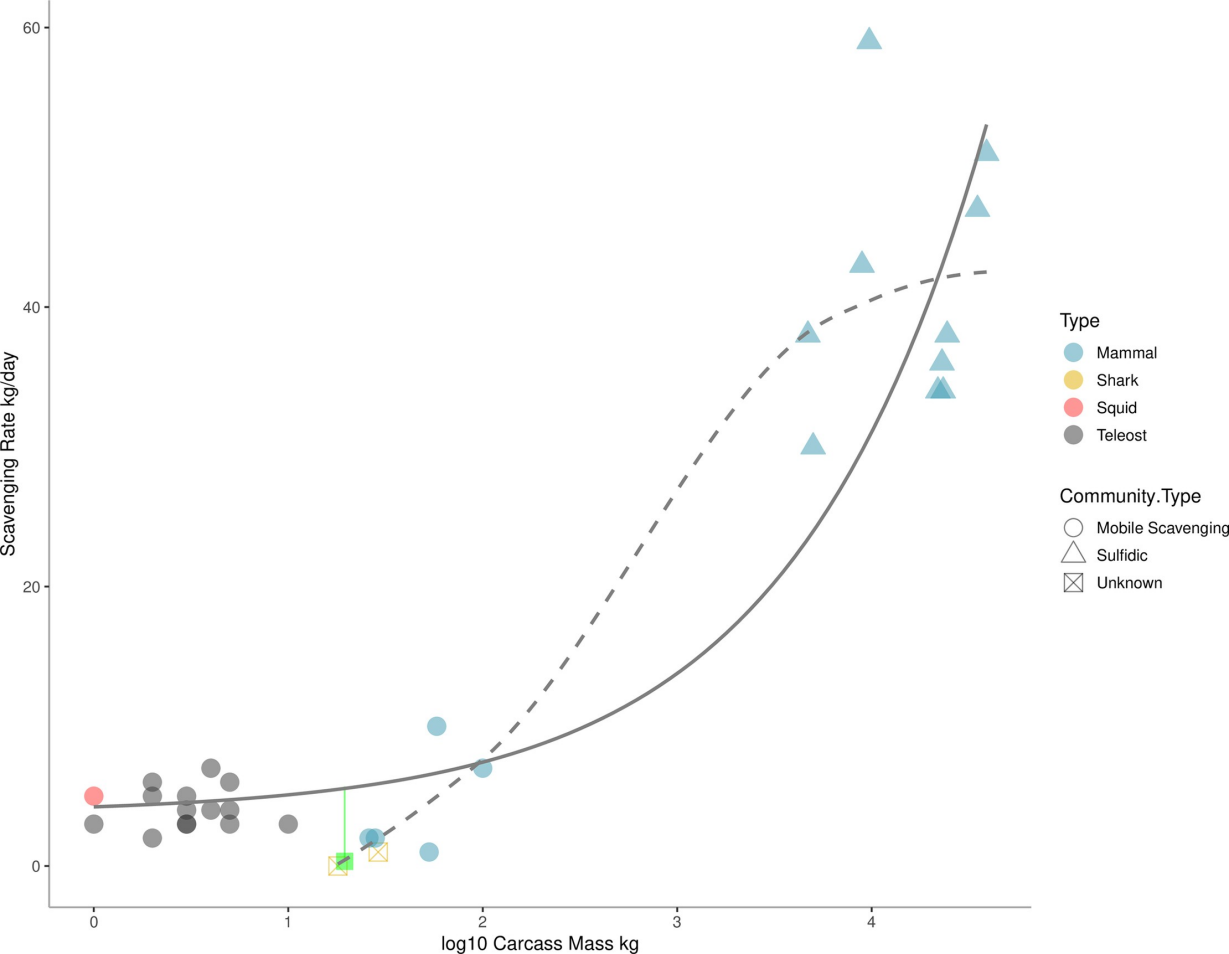
Scavenging rates (kg per day) versus total mass (kg) of animal falls in the deep sea. Point color denotes animal type. Circles denote animal falls that reached mobile scavenger stage and triangles those animal falls that reached sulfophilic stages. Green square represents *A*. *mississippiensis* from this study. The base of the green line represents the scavenging rate if *A*. *mississippiensis* had been consumed in 3 days. The solid line represents an exponential regression fit for food falls and the dashed line the Loess smoothed fit for just non-teleost communities. Data originally from Higgs et al. [28].

All three of the deployed alligators in this study were certainly in the mobile-scavenger stage, though scavenger assemblage differed across the individual carcasses. Besides *Osedax*, all species are known from the background. It is possible that carcass A2 was in the early stages of the enrichment-opportunistic stage when observed at 51 days. There was no visual sign of a change in sediment community or presence of bacterial mat. However, the sediment around the bones was clearly disturbed. Upon collection and closer inspection, the sediment was found to be finer in samples closer to the bones than in background samples. Additionally, the sediment collected around the bones smelled of decaying organic matter. This may suggest organic enrichment within the sediments, and further identification of macrofauna within the samples will reveal if there is a clear change is the macrofaunal community assemblage. The alligator falls in this experiment may not have reached later sulfophilic successional stages due the size of the carcass being below some critical threshold (Fig 6), as noted in previous research [6, 28]. Other smaller food falls fail to form either the enrichment-opportunist or sulfophilic stages. Clear sulfophilic stages are only known from whales ~3000 kilograms or over. Alternatively, the lipid content may have been insufficient for the late stage chemosynthetic community to develop [6, 28]. Given that lipid content of Nile crocodile bone marrow is between 51–68% [57] and whale bones can be up to 60% lipid in terms of weight there appears to be little difference in the quality of whale and crocodilian bones. The lack of this successional stage may be driven by sheer mass of lipids contained in whale bones.

Here, we present the first observations of *Osedax* from the Gulf of Mexico. These results support other studies that have found that bones of organisms beyond whales can support *Osedax* [58, 59]. This is the first published report of *Osedax* exploiting the bones of Crocodilia, though three species (*O*. *knutei*, *O*. *ryderi*, and *O*. *talkovici*) have been recovered from green turtle bones deployed off California. These three *Osedax* species also exploited the bones of other taxa ranging from teleosts to mammals and there is no evidence to date of specialization on any particular kind of bone [54]. The new species of *Osedax* found here may therefore not be an alligator-specialist, but notably it was not found colonizing cow bones deployed at a similar depth in the Gulf of Mexico. The Alligator 2 bones were recovered after 51 days of deployment and had maturing *Osedax* females. While this seems to be a very rapid time from recruitment to maturity, *Osedax* is known for such quick colonization once bones are exposed [60]. The lack of obvious dwarf males may be artefactual owing to the preservation method of the bones (ethanol), which rendered the tubes opaque and males difficult to discern. The clade to which the new species belongs (Clade II or ‘nudepalps’) is widely distributed from California to Japan to Antarctica and there is presently no obvious biogeographic pattern to its discovery in the Gulf of Mexico. The geographically most proximate known *Osedax* are from the northern Atlantic (*O*. *mucofloris*) and off Brazil (*O*. *braziliensis*) [61], but these are phylogenetically quite distant in belonging to *Osedax* clade IV [54].

The complete absence of the A3 individual after 8 days suggest a large scavenger consumed the alligator fall. Additionally, the clear evidence of 20.4 kg anchor weight for the food fall being moved 8.3 meters away implies a larger-sized organism. Given the depth of the alligator fall (~2000m) and implied body size necessary to both consume a moderately-sized alligator and move a large weight, we posit that a large shark may have consumed the alligator fall. Sleeper sharks, *Somniosus* (*Somniosus*) sp., possess a biogeographic range encompassing the Gulf of Mexico, often associate with the bottom at depths of up to 2400 meters, and reach considerable sizes (Greenland shark > 7m) [62]. Video evidence and a caught specimen also indicate that Greenland sharks feed on marine mammals, their carcasses as well as the carcasses of terrestrial animals [63–65]. Alternatively, sixgill sharks (*Hexanchus* sp.) are also known from the Gulf of Mexico at these depths (~2500m) and reach sizes near 5 meters [62]. Sixgills are known to feed on a wide range of marine organisms include large pinnipeds [66]. In 2013, during filming for the Shark Week episode "Alien Sharks", bluntnose sixgill sharks were even found to be territorial, laying claim to the carcass of a sperm whale calf used to lure deep-sea shark species within range of submarine cameras. One other potential predator is the ragged-tooth shark (*Odontaspis ferox)*, which has been observed in the Gulf of Mexico at over 500 m and is thought to occur deeper [67].

The findings of this experiment indicate that terrestrial and aquatic-based carbon in the form of large reptiles is accessible and utilized in deep marine systems. The importance of reptile falls for the carbon cycling and the deep ocean is unknown but could potentially be a global and large impact. Reptile falls may be locally concentrated in areas with major river drainages, e.g. Mississippi, Amazon, and Nile, but geographically broad. The distribution of large crocodilians ranges throughout the tropics and extends into the temperate climates of south-eastern United States and the Yangtze River in China [37]. Of the 24 species, all range from 1.5–7 meters in length providing a considerable source of carbon for marine systems [37]. With a total population over 1,000,000 [37], assuming an average weight of 90kg (typical for adult females), water content of 20% [68], and a carbon content of 47% [69, 70], then the American alligator represents a carbon store of 7,920 metric tons. If we assume the average community at 2km depth respires 29.10 mg C m^-2^ d^-1^ [71], and the area around an alligator fall is 2 m^2^, then a single alligator represents 136 days of respiration. If only 1% of the alligator population becomes deep-sea food falls, this would represent the equivalent of 7,456 years’ worth of carbon respiration for a 1 m^2^ patch or ¾ of hectare area for a year.

Scavenging rates for alligator falls are comparable to rates from other studies’ similarly-sized animal falls (Fig 6). Previous research posited that tough skin of elasmobranchs composed of dermal denticles may inhibit initial scavenging [28]. Alligator skin is cornified and is clad in non-overlapping scales, or scutes, that would also produce a tough and thick barrier for scavengers. However, the alligator fall was quickly detected and utilized by scavengers. Observations of these alligator falls also indicate that the impact of a food fall may be not only from enrichment but through disturbance as well. Disturbance regimes are known to alter macrofauna composition and diversity [72] and similar regime may be occurring at food falls as suggested by our observations. Last, the variability in outcomes in two of the alligator falls—one consumed by many smaller benthic scavengers and another potentially taken by single larger benthopelagic scavenger—highlight that carbon from large food falls may enter deep-sea food webs through different pathways.

## Supporting information

S1 Fig Maximum likelihood tree based on COI sequences for all available species of *Osedax* on Genbank, plus the *Osedax* recorded here from the Gulf Mexico Alligator fall (arrow). GenBank accession numbers are listed with the name of the terminal. The new species falls among what is known as Clade II of Osedax, which are distinguished in having ‘nudepalp's. See Rouse et al. (2018). Branch lengths are indicative of genetic distance and show the *Osedax* from the Alligator fall is clearly a new species.
(PDF)
