# Supplementary figures and images for: Alligators in the abyss: The first experimental reptilian food fall in the deep ocean
Source: PLoS One. 2019 Dec 20;14(12):e0225345. doi: 10.1371/journal.pone.0225345 (PMC6924670; doi:10.1371/journal.pone.0225345)

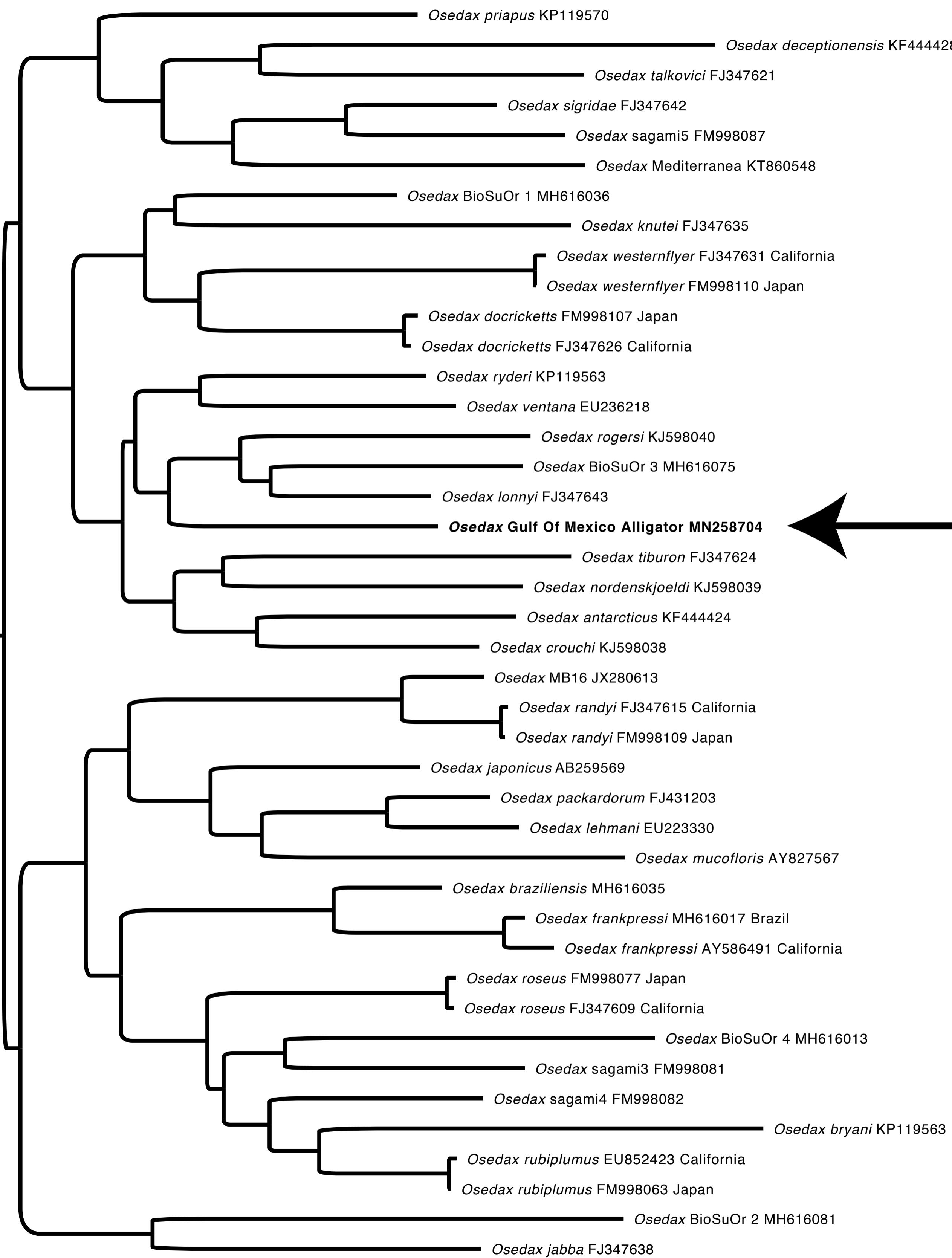

0.04

Supplement: S1 Fig — GenBank accession numbers are listed with the name of the terminal. The new species falls among what is known as Clade II of Osedax, which are distinguished in having ‘nudepalp's. See Rouse et al. (2018). Branch lengths are indicative of genetic distance and show the Osedax from the Alligator fall is clearly a new species. (PDF) [file pone.0225345.s001.pdf]
